# Identification of Potent and Safe Antiviral Therapeutic Candidates Against SARS-CoV-2

**DOI:** 10.3389/fimmu.2020.586572

**Published:** 2020-11-25

**Authors:** Xia Xiao, Conghui Wang, De Chang, Ying Wang, Xiaojing Dong, Tao Jiao, Zhendong Zhao, Lili Ren, Charles S. Dela Cruz, Lokesh Sharma, Xiaobo Lei, Jianwei Wang

**Affiliations:** ^1^ NHC Key Laboratory of System Biology of Pathogens, Institute of Pathogen Biology, Chinese Academy of Medical Sciences & Peking Union Medical College, Beijing, China; ^2^ Key Laboratory of Respiratory Pathogenomics, Chinese Academy of Medical Sciences, Beijing, China; ^3^ Christophe Merieux Laboratory, Institute of Pathogen Biology, Chinese Academy of Medical Sciences & Peking Union Medical College, Beijing, China; ^4^ The Department of Pulmonary and Critical Care Medicine, Third Medical Center of Chinese PLA General Hospital, Beijing, China; ^5^ Section of Pulmonary and Critical Care and Sleep Medicine, Department of Medicine, Yale University School of Medicine, New Haven, CT, United States

**Keywords:** SARS-CoV-2, COVID-19, antiviral, therapy, US Food and Drug Administration-approved compounds

## Abstract

COVID-19 pandemic has infected millions of people with mortality exceeding >1 million. There is an urgent need to find therapeutic agents that can help clear the virus to prevent severe disease and death. Identifying effective and safer drugs can provide more options to treat COVID-19 infections either alone or in combination. Here, we performed a high throughput screening of approximately 1,700 US FDA-approved compounds to identify novel therapeutic agents that can effectively inhibit replication of coronaviruses including SARS-CoV-2. Our two-step screen first used a human coronavirus strain OC43 to identify compounds with anti-coronaviral activities. The effective compounds were then screened for their effectiveness in inhibiting SARS-CoV-2. These screens have identified 20 anti-SARS-CoV-2 drugs including previously reported compounds such as hydroxychloroquine, amlodipine besylate, arbidol hydrochloride, tilorone 2HCl, dronedarone hydrochloride, mefloquine, and thioridazine hydrochloride. Five of the newly identified drugs had a safety index (cytotoxic/effective concentration) of >600, indicating a wide therapeutic window compared to hydroxychloroquine which had a safety index of 22 in similar experiments. Mechanistically, five of the effective compounds (fendiline HCl, monensin sodium salt, vortioxetine, sertraline HCl, and salifungin) were found to block SARS-CoV-2 S protein-mediated cell fusion. These FDA-approved compounds can provide much needed therapeutic options that we urgently need during the midst of the pandemic.

## Introduction

Novel coronavirus (CoV)–mediated disease (COVID-19) emerged as a major pandemic and has spread across the world in such a short period since December 2019. As of October 5, 2020, more than 35.1 million confirmed infections have been reported with approximately 1 million deaths (WHO, https://covid19.who.int/). These numbers may be a vast underestimation as many of the infected patients may remain asymptomatic and can only be detected by antibody testing (1). Similarly, many of the deaths may not be accounted for due to a lack of testing. The disease is caused by a novel CoV termed SARS-CoV-2 which belongs to the *Coronaviridae* family and is the third major CoV pandemic in the last 20 years after Severe Acute Respiratory Syndrome (SARS) and Middle East Respiratory Syndrome (MERS) (2–7). The lack of available therapeutic options is a major limiting factor in treating these infections, leading to excessive mortality.

Currently, there is an urgent and unmet need for effective antiviral therapy that can not only decrease the disease burden in the patient but can also decrease the ability of the person to infect others. It is not practical to develop a novel drug for urgent needs such as during the current pandemic, which may take years to confirm safety and efficacy. Alternatively, it sounds like a lucrative option to repurpose the US Food and Drug Administration (FDA) approved drugs for their efficacy against SARS-CoV-2. Earlier screens have found antiviral efficacy of approved therapies such as hydroxychloroquine (8–14); however, these therapies failed to provide any beneficial effects in COVID-19 due to their toxic side effects (13–20). Finding efficacy of an approved drug against SARS-CoV-2 with minimal toxicity can provide much needed therapeutic option to treat COVID-19.

Here we screened approximately 1,700 US FDA-approved compounds to test their ability to inhibit SARS-CoV-2 replication. Here, we report 20 compounds that are highly effective in inhibiting SARS-CoV-2 replication at concentrations that were significantly lower than those having cytotoxic effects. We also investigated the possible mechanism of these compounds.

## Materials and Methods

### Cells and Viruses

LLC-MK2 cells (Rhesus monkey kidney cells), which was provided by the Laboratoire des Pathogènes Emergents (LPE), Fondation Mérieux, Lyon, France, were cultured in 64% Hank’s MEM and 32% Earle’s MEM (Gibco, New York, USA) supplemented with 3% fetal bovine serum (FBS) (Hyclone, Utah, USA) and 1% glutamine (Thermo, Massachusetts, USA). Vero cells (African green monkey kidney cell), purchased from ATCC, were cultured in Dulbecco’s Modified Eagle’s Medium (DMEM, Gibco) supplemented with 10% FBS.

Human coronavirus (HCoV) strain OC43, which is a gift from Peking Union Medical College Hospital, was propagated in LLC-MK2 cells in 0.5% FBS MEM and virus titers were determined *via* TCID50 with LLC-MK2 cells. SARS-CoV-2 virus was isolated from the respiratory samples of patients in Wuhan of Hubei Province (3). SARS-CoV-2 virus was propagated in Vero cells and used in this study. All experiments with SARS-CoV-2 virus were conducted in a BSL-3 laboratory.

HEK293T cells stably expressing recombinant human ACE2 (293T/hACE2) were maintained in Dulbecco’s MEM containing 10% FBS and 100 units penicillin, and 100 μg of streptomycin per milliliter.

### Antibodies

Mouse polyclonal against the OC43 N antibody was prepared in the laboratory. Rabbit polyclonal against SARS-CoV-2 N protein antibody was purchased from Sino Biological (Beijing, China). Alexa Fluor 488–conjugated goat anti-mouse IgG, Alexa Fluor 488–conjugated goat anti-rabbit IgG were purchased from Thermo.

### Screening of FDA-Approved Drugs

US FDA-approved drug library which contains 1,700 compounds were purchased from TargetMol (Massachusetts, USA). LLC-MK2 cells were seeded at 2 × 10^4^ cells per well in 96-well plates and incubated at 37°C and 5% CO_2_. The next day, LLC-MK2 cells were treated with the compounds at a concentration of 10 μM. After 1 h of treatment, cells were infected with OC43 at MOI of 1. At 48 h post-infection (hpi), cells were fixed with 4% paraformaldehyde for 20 min at room temperature. Immunofluorescence staining was performed using mouse anti-OC43 NP antibody, followed by anti-mouse Alexa Fluor 488 and DAPI (Sigma, St. Louis, MO). Images were captured by Operetta (PerkinElmer, Massachusetts, USA) at the magnification of 20× objective. The infection ratios were calculated using automated image analysis software (Harmony 3.5.2, PerkinElmer). Remdesivir and DMSO were used as positive and negative controls, respectively.

The positively identified drugs from this screen were used to perform dose-response curves against OC43 on LLC-MK2 and against SARS-CoV-2 using Vero cells as described below.

### IC_50_ (the Half-Maximal Inhibitory Concentration), CC_50_ (the Half-Maximal Cytotoxic Concentration), and SI (Selectivity Index) Determination

LLC-MK2 cells (for OC43 infection) or Vero cells (for SARS-CoV-2 infection) were seeded in 96 wells plate one day before infection at the concentration of 2 × 10^4^ cells/well or 1.4 × 10^4^ cells/well, respectively. For IC_50_, cells were pre-treated for 1 h with each drug at concentrations 0.013, 0.041, 0.123, 0.370, 1.111, 3.333, 10, and 30 μM and then infected with the virus at MOI of 1. Cell culture media was then replaced containing same concentration of the drug to ensure presence of drug at the time of infection. At 48 hpi (OC43) or 24 hpi (SARS-CoV-2), cells were fixed with 4% paraformaldehyde for 20 min at room temperature. Immunofluorescence was conducted with mouse anti-OC43 N protein antibody or rabbit anti-SARS-CoV-2-NP antibody and followed by anti-mouse, or anti-rabbit Alexa Flour 488 and DAPI. Images were performed by Operetta with 20× objective. The IC_50_ was calculated using automated image analysis software (Harmony 3.5.2, PerkinElmer).

For CC_50_, cells were pre-treated with each drug at concentrations 0.013, 0.041, 0.123, 0.370, 1.111, 3.333, 10, and 30 μM, respectively. After 48 h (LLC-MK2 cells) or 24 h (Vero cells) post-treatment, cell viability was evaluated by using a CCK8 kit (Yeasen, Beijing, China) according to the manufacturer’s instructions. The selectivity index was calculated using the following formula: SI = CC_50_/IC_50._ Graphpad Prism 7.0 was used for analyzing IC_50_ and CC_50_.

### Immunofluorescence

Cells were fixed with 4% paraformaldehyde for 20 min at room temperature, and permeabilized with 0.5% Triton X-100 for 10 min. Cells were then blocked with 5% BSA and stained with primary antibodies, followed by staining with an Alexa Fluor 488 secondary antibodies. Nuclei were counterstained with DAPI.

### Quantitative RT-PCR

Vero cells were pre-treated with indicated concentrations of drugs for 1 h and incubated with SARS-CoV-2 at 0.1 MOI for 1 h. Then, cells were washed with opti-MEM for one time and incubated with indicated concentrations of drugs. At 24 hpi, supernatants were collected and viral RNA in the cell supernatants were extracted by using Direct-zol RNA MiniPrep kit (Zymo Research, CA, USA) according to the manufacturer’s instructions. Viral copy numbers were measured by RT-PCR using primers and probe targeting the SARS-CoV-2 N gene. The reference standard was tenfold diluted from 1 × 10^9^ copies to 1 × 10^4^ copies. PCR amplification cycle was 50°C, 15 min, 95°C, 3 min; 95°C, 15 s, 60°C, 45 s+ Plate Read, 50 cycles. The amplification process, fluorescence signal detection, data storage, and analysis were all completed by quantitative fluorescence PCR and its own software (Bio-Rad CFX Manager). The copies of the virus were calculated according to the standard curve. The inhibition ratio was obtained by dividing the number of copies of the virus in the vehicle control group. The data were nonlinearly fitted by GraphPad 7.0 software to calculate the IC_50_ of each drug.

### Cell-Cell Fusion Assay

Cell-cell fusion assays were performed as described previously (20). Briefly, HEK-293T cells were co-transfected with SARS-CoV-2-S glycoprotein and eGFP. At 24 h post-transfection, cells were digested with trypsin (0.25%) and overlaid on a 50% confluent monolayer of 293T-ACE2 cells at a ratio of 1:1 which were treated with candidate drugs for 1 h. After 24 h incubation, syncytial images were captured with Operetta (PerkinElmer, Massachusetts, USA).

## Results

### Inhibitory Potential of FDA-Approved Drugs Against hCoV OC43

**Figure 1:**
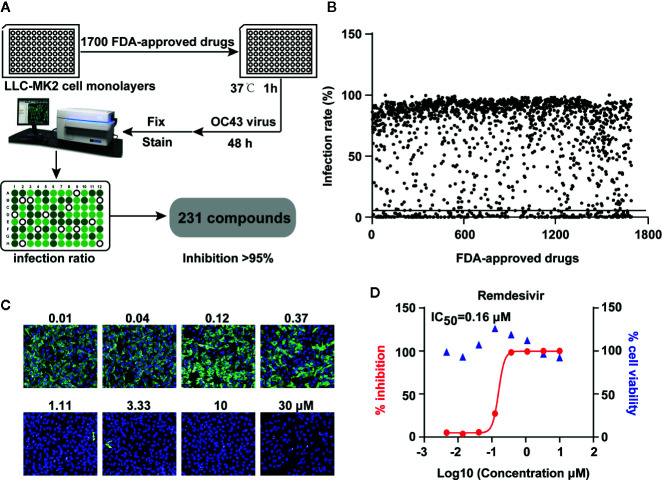
High-throughput screening of US FDA-approved drug library to inhibit human coronavirus OC43 replication *in vitro*. **(A)** The strategy of high throughput screening to identify antiviral drugs that effectively inhibit OC43 replication. LLC-MK2 cells were pretreated with FDA-approved drugs at 10 μM for 1 h and infected with 1 MOI of OC43 for 48 h. Cells were then fixed and stained to calculate the infection ratio with Operetta software. **(B)** Primary screening results of 1,700 FDA-approved drugs against OC43, each dot represents one compound along with the rate of OC43 inhibition. **(C)** Image samples show signals of OC43 infection in cell cultures. LLC-MK2 cells were treated with indicated doses of remdesivir for 1 h, and then cells were infected with OC43 for 48 h. **(D)** Cell viability of remdesivir to LLC-MK2 cells were measured by CCK-8 assays. The % inhibitions were calculated according to the data in **(C)**.

Initial screening was performed using HCoV-OC43 due to its low biosafety concerns. OC43 is a hCoV that usually causes mild disease in humans and cattle (21). The experimental protocol is demonstrated in **Figure 1A** using LLC-MK2 cells infected with OC43 at an MOI of 1 for 48 h in presence of the US FDA-approved compounds. The inhibitory potentials of these compounds were measured with the treatment of compounds at 10 μM for 48 h. The viral presence was detected by immunostaining for the virus and DAPI staining for the cell nuclei. The inhibitory capacity was measured using the ratio of viral fluorescence to the DAPI and is depicted in **Figure 1B**. The initial screen obtained 231 compounds that had the ability to inhibit OC43 replication >95%. The remdesivir was used as a positive control (**Figures 1C, D**).

### Calculation of IC_50_, CC_50,_ and SI of FDA-Approved Drugs Against OC43

**Figure 2:**
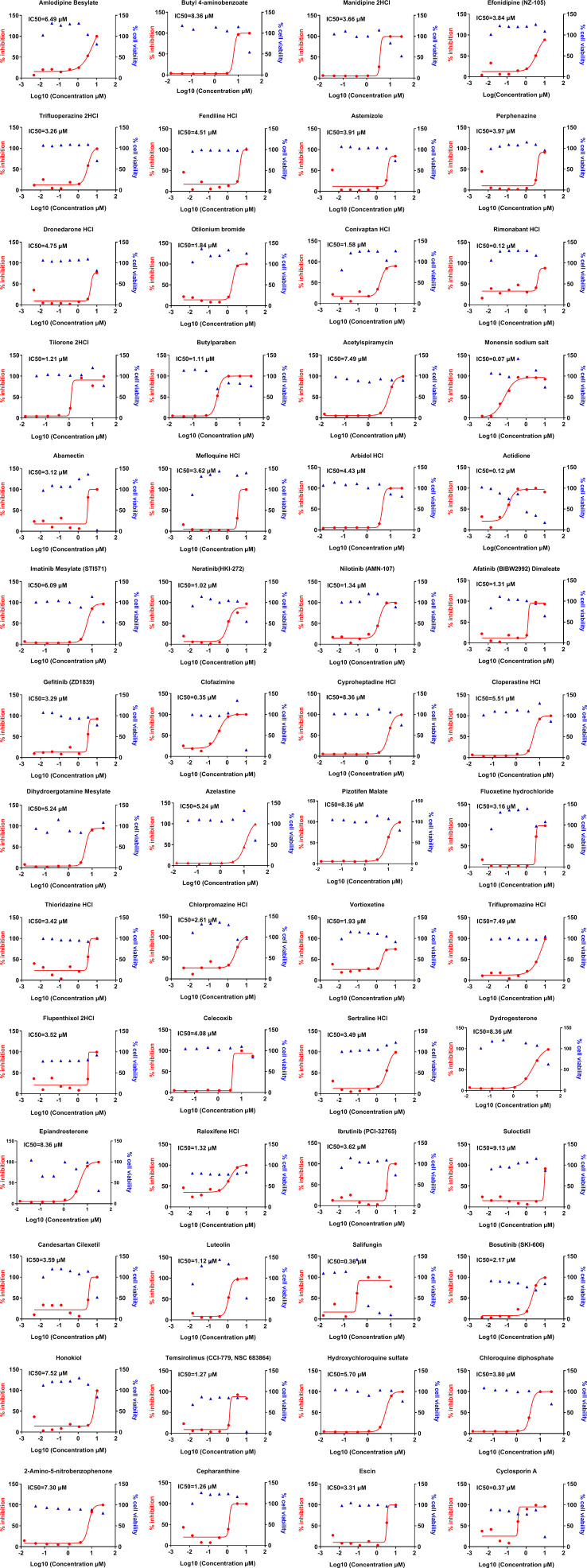
Dose-response curves of selected compounds from the hits against OC43 infection *in vitro*. LLC-MK2 cells were pretreated with indicated drugs at 37°C for 1 h with eight doses (0.014, 0.041, 0.123, 0.370, 1.111, 3.333, 10, 30 μM) with three-fold dilution followed by infection with OC43 at MOI of 1 for 48 h. In parallel, these compounds’ effects on the cell viability in LLC-MK2 cells were measured by CCK-8 assays. The left *Y*-axis of the graphs represents % inhibition of the infection (red dots) and the right *Y*-axis of the graphs presents % cell viability (blue triangles) in the presence of the drugs.

Next, we sought to determine the effective concentrations of positively screened drugs in our initial approach and test the toxicity profile of these 231 drugs in relation to their viral inhibitory concentrations. Our data show that 56 of the positively screened drugs were effectively inhibited the viral replication at sub-micromolar concentrations including many of them can almost completely inhibit the viral replication at micromolar range (**Figure 2**). Surprisingly, the effective drugs against the CoV belonged to a wide range of therapeutic groups including those used for neurological diseases, hormones, enzymes, and antimicrobial agents among others (**Table 1**). The cytotoxic concentration to kill 50% of cells (CC_50_) was noted for these drugs by measuring cell viability over similar concentrations. The selective index in our study was found to be >600 for 5 of the screened compounds. The SI for the hydroxychloroquine was 22 in our study, indicating increased safety of newly identified drugs compared to the hydroxychloroquine.

**Table 1:** Antiviral activity of selected compounds against OC43.

Pathway	MOLENAME	Formula	Target	CC_50_ (μM)	IC_50_ (μM)	SI (CC_50_/IC_50_)
Membrane Transporter/Ion Channel	Amlodipine Besylate	C_26_H_31_ClN_2_O_8_S	Carbonic anhydrase inhibitor; Calcium Channel inhibitor; PDE inhibitor	28.9	6.49	4.45
	Butyl 4-aminobenzoate	C_11_H_15_NO_2_	Sodium Channel inhibitor	>30	8.36	>3.59
	Manidipine 2HCl	C_35_H_40_Cl_2_N_4_O_6_	Calcium Channel antagonist	>30	3.66	>8.20
	Efonidipine(NZ-105)	C_34_H_38_N_3_O_7_P	Calcium Channel inhibitor	>10	3.84	>2.60
	Trifluoperazine 2HCl	C_21_H_26_Cl_2_F_3_N_3_S	Dopamine Receptor; Adrenergic Receptor antagonist; Calmodulin inhibitor	>10	3.26	>3.08
	Fendiline HCl	C_23_H_26_ClN	Calcium Channel inhibitor	>10	4.51	>2.22
	Astemizole	C_28_H_31_FN_4_O	Histamine Receptor; Potassium Channel inhibitor	>10	3.91	>2.52
	Perphenazine	C_21_H_26_ClN_3_OS	Calmodulin inhibitor; Dopamine Receptor antagonist	>10	3.97	>2.52
	Dronedarone HCl	C_31_H_44_N_2_O_5_S.HCl	Potassium Channel inhibitor; Sodium Channel inhibitor; Adrenergic Receptor antagonist; Calcium Channel inhibitor	>10	4.75	>2.11
	Otilonium bromide	C_29_H_43_BrN_2_O_4_	Calcium Channel inhibitor; AChR antagonist	>10	1.84	>5.43
GPCR/G Protein	Conivaptan HCl	C_32_H_26_N_4_O_2_.HCl	Vasopressin Receptor antagonist	>10	1.58	>6.33
	Rimonabant HCl	C_22_H_21_Cl_3_N_4_O	Cannabinoid Receptor	>10	4.11	>2.43
Microbiology & Virology	Tilorone 2HCl	C_25_H_34_N_2_O_3_	Antiviral	>30	1.21	>24.79
	Butylparaben	C_11_H_14_O_3_	Antibacterial	>30	1.11	>27.03
	Acetylspiramycin	C_45_H_76_N_2_O_15_	Antibiotic	>30	7.49	>4.00
	Monensin sodium salt	C_36_H_61_NaO_11_	Antibiotic	>10	0.07	>142.86
	Abamectin	C_49_H_74_O_14_	Antiparasitic	~10	3.12	~3.21
	Mefloquine HCl	C_17_H_17_ClF_6_N_2_O	Hemozoin synthesis inhibitor	12.52	3.62	3.46
	Arbidol HCl	C_22_H_25_BrN_2_O_3_S.HCl	Influenza Virus	8.85	4.43	2.00
	Actidione	C_15_H_23_NO_4_	Antifungal	1.77	0.12	14.75
Tyrosine Kinase/Adaptors	Imatinib Mesylate (STI571)	C_29_H_31_N_7_O.CH_4_SO_3_	Bcr-Abl inhibitor; c-kit inhibitor; PDGFR inhibitor	>30	6.09	>4.93
	Neratinib(HKI-272)	C_30_H_29_ClN_6_O_3_	EGFR inhibitor; HER2 inhibitor	>10	1.02	>9.80
	Nilotinib (AMN-107)	C_28_H_22_F_3_N_7_O	Bcr-Abl inhibitor; Mast/stem cell GFR Kit agonist	>10	1.34	>7.46
	Afatinib (BIBW2992) Dimaleate	C_32_H_33_ClFN_5_O_11_	EGFR inhibitor; HER2 inhibitor	>10	3.62	2.76
	Gefitinib (ZD1839)	C_22_H_24_ClFN_4_O_3_	EGFR antagonist	>10	3.29	>3.34
Enzyme	Clofazimine	C_27_H_22_Cl_2_N_4_	CzcO-like inhibitor; DNA intercalation	~10	0.35	~28.57
Neuroscience	Cyproheptadine HCl	C_21_H_22_ClN	Histamine Receptor antagonist; 5-HT receptor antagonist; AChR antagonist	>30	8.36	>3.59
	Cloperastine HCl	C_20_H_25_Cl_2_NO	Histamine Receptor antagonist	>30	5.51	>5.44
	Dihydroergotamine Mesylate	C_34_H_41_N_5_O_8_S	5-HT Receptor antagonist	>30	5.24	>5.73
	Azelastine	C_22_H_25_Cl_2_N_3_O	Histamine Receptor antagonist	>30	12.01	>2.50
	Pizotifen	C_19_H_21_NS	5-HT Receptor antagonist	>30	8.36	>3.59
	Fluoxetine hydrochloride	C_17_H_18_F_3_NO.HCl	5-HT Receptor antagonist	>10	3.16	>3.16
	Thioridazine HCl	C_21_H_27_ClN_2_S_2_	5-HT Receptor antagonist; Adrenergic Receptor antagonist; Dopamine Receptor antagonist; Potassium Channel inhibitor	>10	3.42	>2.92
	Chlorpromazine HCl	C_17_H_19_ClN_2_S.HCl	Histamine Receptor antagonist; Potassium Channel inhibitor; Dopamine Receptor antagonist; Adrenergic Receptor antagonist; AChR antagonist; 5-HT receptor antagonist	>10	2.61	>3.83
	Vortioxetine	C_18_H_22_N_2_S	5-HT Receptor modulator	>10	1.93	>5.18
	Triflupromazine HCl	C_18_H_20_ClF_3_N_2_S	Dopamine Receptor antagonist; AChR antagonist; 5-HT receptor antagonist	>10	7.49	>1.34
	Flupenthixol 2HCl	C_23_H_27_Cl_2_F_3_N_2_OS	Dopamine Receptor antagonist	>10	3.52	>2.84
	Celecoxib	C_17_H_14_F_3_N_3_O_2_		29.83	4.08	7.31
	Sertraline HCl	C_17_H_17_CI_2_N.HCl	5-HT Receptor antagonist	>10	3.49	>2.87
Endocrinology/Hormones	Dydrogesterone	C_21_H_28_O_2_	Progesterone Receptor agonist	>30	8.36	>3.59
	Epiandrosterone	C_19_H_30_O_2_	Androgen Receptor agonist	>30	8.36	>3.59
	Raloxifene HCl	C_28_H_28_ClNO_4_S	Estrogen/Progestogen Receptor antagonist; SERM agonist	>10	1.32	>7.58
Angiogenesis	Ibrutinib (PCI-32765)	C_25_H_24_N_6_O_2_	BTK inhibitor	>10	3.62	>2.76
	Suloctidil	C_20_H_35_NOS	Antiplatelet aggregation inhibitor	>10	9.13	>1.10
	Candesartan Cilexetil	C_33_H_34_N_6_O_6_	RAAS antagonist; ACE inhibitor	~10	3.59	~2.79
Others	Luteolin	C_15_H_10_O_6_	TNF-alpha inhibitor, IL inhibitor; NF-κB inhibitor	3.5	1.12	3.13
	Salifungin	C13H9BrClNO2	Others	1.08	0.36	3.00
	Bosutinib (SKI-606)	C_26_H_29_Cl_2_N_5_O_3_	MAPK inhibitor; Src inhibitor; Bcr-Abl inhibitor; CaMK inhibitor; CDK inhibitor	>10	2.17	>4.61
	Honokiol	C_18_H_18_O_2_	Akt inhibitor; MEK inhibitor	>10	7.52	>1.33
	Temsirolimus (CCI-779, NSC 683864)	C_56_H_87_NO_16_	mTOR inhibitor	9.3	1.27	7.32
	Hydroxychloroquine sulfate	C_18_H_26_ClN_3_O.H_2_SO_4_	Autophagy inhibitor; TLR antagonist	>30	5.7	>5.26
	Chloroquine diphosphate	C_18_H_26_CLN_3_.2(H_3_PO_4_)	Autophagy inhibitor; ATM/ATR activator	>30	3.8	>7.89
	2-Amino-5-nitrobenzophenone	C_13_H_10_N_2_O_3_	Intermediates	>30	7.3	>4.11
	Cepharanthine	C_37_H_38_N_2_O_6_	Others	9.1	1.26	7.22
	Escin	C_55_H_86_O_24_	Others	>10	3.31	>3.02
	Cyclosporin A	C_62_H_111_N_11_O_12_	Others	~10	0.37	~27.03

### Testing the Antiviral Effectiveness of Positive Drugs Against SARS-CoV-2

**Figure 3:**
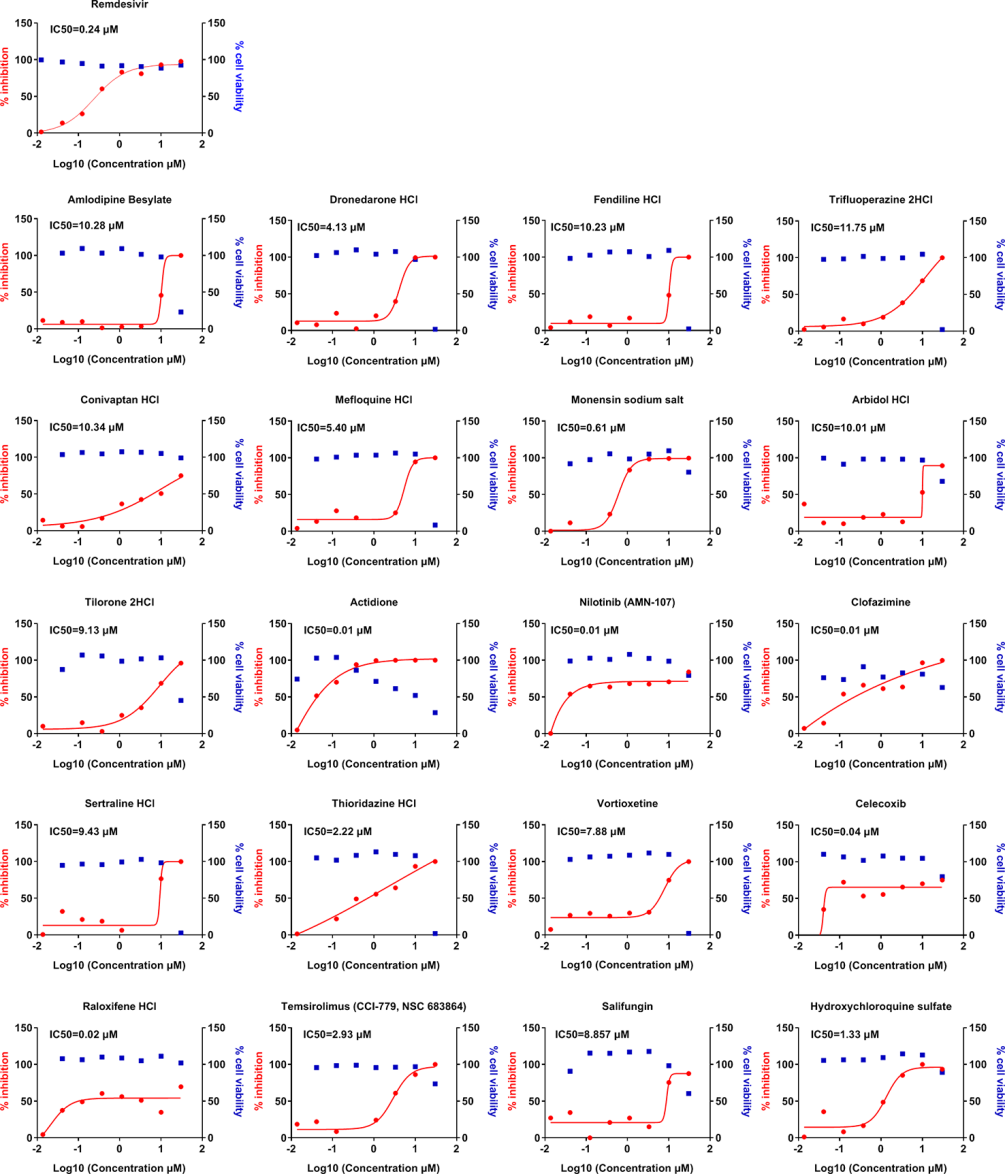
Dose-response curves of selected compounds against SARS-CoV-2 infection. Vero cells were pretreated with indicated drugs at 37°C for 1 h with eight doses (0.014, 0.041, 0.123, 0.370, 1.111, 3.333, 10, and 30 μM) with three-fold dilutions, followed by infection with SARS-CoV-2 at an MOI of 0.1 for 24 h. The viral load in the cell supernatant was quantified by qRT-PCR. Meanwhile, cell viability in the presence of these drugs was measured in Vero cells by CCK-8 assays. The left *Y*-axis of the graphs represents % inhibition of viral replication (red dots) and the right *Y*-axis of the graphs indicates % cell viability (blue squares) in the presence of the drugs.

Finally, we sought to determine whether these 56 compounds can show efficacy against SARS-CoV-2, the causative agent of COVID-19. These 56 compounds from the initial screen were tested for their antiviral efficacy against SARS-CoV-2 in Vero cells. SARS-CoV-2 replicates within the Vero cells and causes cytopathic effects in these cells in the absence of any antiviral treatment. We generated the dose-response inhibition curves along with the cytotoxicity curves for these compounds in the presence of SARS-CoV-2 (**Figure 3**). Remdesivir was used as a positive control. Our data show that 20 of these compounds show significant efficacy in inhibiting SARS-CoV-2 replication with sub micromolar IC_50_ for many of these drugs such as nilotinib, clofazimine, and raloxifene. The effects also confirmed by immunofluorescence assay (data not shown). These compounds also belong to a wide variety of classes including cardiac glycosides, anti-malarial drug hydroxychloroquine, cyclooxygenase-2 inhibitors, and ion channel blockers. The IC_50_, CC_50_, and SI of these compounds are shown in **Table 2**.

**Table 2:** Antiviral activity of selected compounds against SARS-CoV-2.

Pathway	MOLENAME	Formula	CC50, IC50(μM)	SI
Membrane Transporter/Ion Channel	Amlodipine Besylate	C_26_H_31_ClN_2_O_8_S	>30, 10.28	>2.92
	Dronedarone HCl	C_31_H_44_N_2_O_5_S.HCl	12.62, 4.13	3.06
	Fendiline HCl	C_23_H_26_ClN	29.24, 10.23	2.86
	Trifluoperazine 2HCl	C_21_H_26_Cl_2_F_3_N_3_S	29.29, 11.75	2.49
GPCR/G Protein	Conivaptan HCl	C_32_H_26_N_4_O_2_.HCl	12.7, 10.34	1.23
Microbiology & Virology	Mefloquine HCl	C_17_H_17_ClF_6_N_2_O	29.13, 5.4	5.39
	Monensin sodium salt	C_36_H_61_NaO_11_	>30, 0.6019	>49.84
	Arbidol HCl	C_22_H_25_BrN_2_O_3_S.HCl	22.36, 10.01	2.23
	Tilorone 2HCl	C_25_H_34_N_2_O_3_	28.67, 9.13	3.14
	Actidione	C_15_H_23_NO_4_	6.06, 0.01	606
Tyrosine Kinase/Adaptors	Nilotinib (AMN-107)	C_28_H_22_F_3_N_7_O	>30, <0.01	>3000
Enzyme	Clofazimine	C_27_H_22_Cl_2_N_4_	>30, 0.01	>3000
Neuroscience	Sertraline HCl	C_17_H_17_CI_2_N.HCl	27.84, 9.34	2.98
	Thioridazine HCl	C_21_H_27_ClN_2_S_2_	27.22, 2.22	12.26
	Vortioxetine	C_18_H_22_N_2_S	28.03 7.88	3.56
	Celecoxib	C_17_H_14_F_3_N_3_O_2_S	>30, 0.04	>750
Endocrinology/Hormones	Raloxifene HCl	C_28_H_28_ClNO_4_S	>30, 0.02	>1500
Others	Temsirolimus (CCl-779, NSC 683864)	C_56_H_87_NO_16_	>30, 2.93	>10.24
	Salifungin	C_13_H_9_BrClNO_2_	11.12, 8.86	1.26
	Hydroxychloroquine sulfate	C_18_H_26_ClN_3_O.H_2_SO_4_	>30, 1.33	>22.56

### Five Candidate Drugs Inhibit Cell Fusion

**Figure 4:**
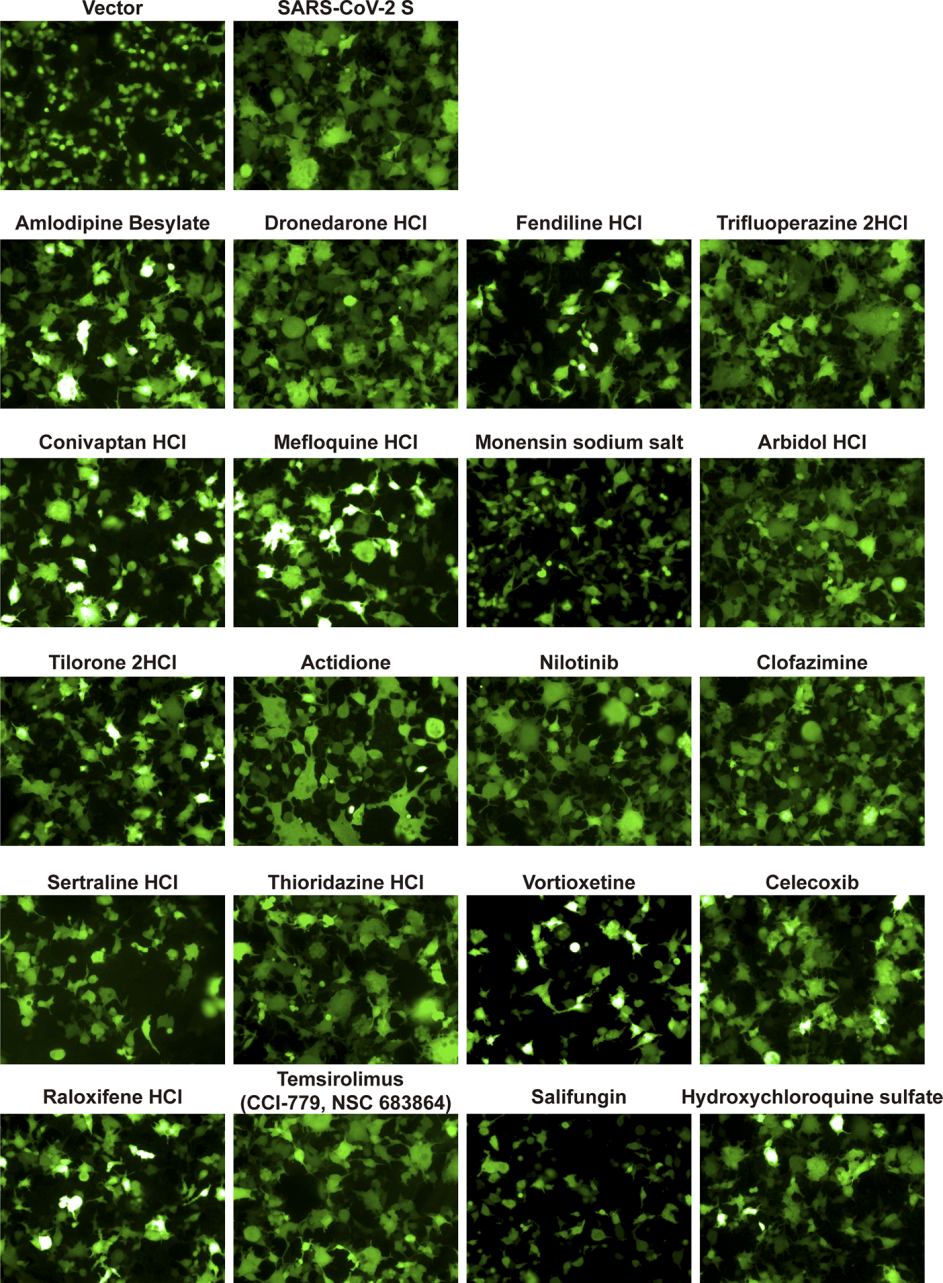
The effect of indicated drugs on cell-cell fusion mediated by SARS-CoV-2 S protein. HEK-293T cells were co-transfected with SARS-CoV-2-S glycoprotein and eGFP. 24 h post-transfection, cells were digested with trypsin (0.25%) and overlaid on a 50% confluent monolayer of 293T-ACE2 cells at a ratio of 1:1 which were treated with candidate drugs at 10 μM for 1 h. After 24-h incubation, images of syncytia were captured with Operetta and analyzed by Harmony software.

Finally, to determine the mechanism by which the compounds inhibit SARS-CoV-2, we investigated these candidates’ effects on virus entry. First, we constructed the cell-cell fusion assays. As indicated in **Figure 4**, SARS-CoV-2 S protein expression can initiate cell fusion with ACE2-overexpressed cells, but the control vector did not. Then, we detected the effects of these indicated drugs on S protein-mediated cell fusion. Indicated drugs were added to cells at 10 μM before the co-incubation of the cells. We found that fendiline hydrochloride, monensin sodium salt, vortioxetine, sertraline hydrochloride, and salifungin inhibited the SARS-CoV-2 S protein-mediated cell fusion (**Figure 4**).

## Discussion

The current pandemic of COVID-19 is the third major outbreak in this century and the largest outbreak of the CoV in known human history. The three novel CoV outbreaks in such a short time are strong indicators of the potential threat posed by CoVs. While most respiratory viral infection research has been focused on influenza viruses that cause a huge burden of seasonal flu and occasionally pandemic outbreaks, CoV is likely to emerge as a similar or more severe pathogen than flu in long term.

Given the scale and devastation of the current COVID-19 outbreak and the persistent threat of CoVs in causing human disease, there is an urgent need to find effective and safe therapies to treat these patients. Currently, there are no approved therapies for CoVs including SARS-CoV-2. The experimental therapies being used with known antiviral agents either show limited efficacy (remdesivir) or have high systemic toxicity (hydroxychloroquine), limiting their usefulness (22–24). Finding new therapies that are effective and safe are urgently needed. In this study, we have identified many FDA-approved therapies that are highly effective against CoVs, including 20 of the effective agents against SARS-CoV-2. This screening confirms previous reports demonstrating anti-SARS-CoV-2 activity of hydroxychloroquine, amlodipine besylate, arbidol hydrochloride, tilorone 2HCl, dronedarone hydrochloride, mefloquine, celecoxib, and thioridazine hydrochloride (8–10, 25–27), while identifying additional 12 drugs. However, there are seven drugs have been reported when our manuscript was underreview (28–37). The underlying mechanisms of viral replication inhibition by these drugs are not clear. It is highly unlikely that these compounds will have similar antiviral mechanisms given the vast structural and pharmacological diversities of the effective antiviral compounds in our study. However, it is clear from other viral studies such as influenza or HIV, where antiviral drugs can affect various viral life cycle steps including attachment, entry, replication, assembly, and budding of viral progeny. Five drugs may inhibit S-mediated cell fusion as indicated by our data (**Figure 4**). Further studies are required to understand the precise mechanisms of each of the effective compounds found in this study.

Toxicity is one of the limiting factors in the therapeutic application of many drugs despite their known antiviral activities. Many of these drugs had SI of >600, showing promise of their usefulness at safe doses. For comparison, the SI of hydroxychloroquine was found to be 22 in our study while SI of amlodipine besylate was found to be ~3, demonstrating a much lower safety profile of this drug. Similarly, other drugs known to have low selective index such as digoxin for their approved use, also show lower SI in our screen. Five of the drugs with SI of >600 include tyrosine kinase inhibitor nilotinib, antibiotics such as clofazimine and actidione, and selective estrogen receptor modulators raloxifene, and non-steroidal anti-inflammatory drug celecoxib.

Betacoronaviruses have raised great public health threats to human beings, as most known HCoVs including all the three virulent HCoVs (SARS-CoV, MERS-CoV, and SARS-CoV-2) and two seasonal HCoVs (OC43 and HKU1) belong to this species (3–7, 21, 38). It is of great value to identify antivirals against a broad spectrum of HCoVs, particularly the Betacoronaviruses, to tackle such threats by pharmaceutical interventions (39, 40). To this end, we first screened the compounds which showed apparent activity of anti-OC43, the most prevalent HCoV circulates worldwide (41). We then narrowed down the candidates by the screening on SARS-CoV-2, resulting in the identification of 20 compounds which can inhibit both OC43 and SARS-CoV-2. Our study provides a foundation for subsequent anti-HCoVs drug screening of a broad spectrum. However, further tests are warranted to verify their efficacies.

In summary, our screen identified 14 previously unknown FDA-approved compounds that are effective in inhibiting SARS-CoV-2 besides confirming the antiviral properties of 7 previously reported compounds, validating our approach. This screen identified five new compounds highly efficacious in inhibiting the viral replication of SARS-CoV-2 with SI >600. Further studies are needed to confirm these drugs’ *in vivo* efficacy in COVID-19 relevant mouse models such as those with human ACE2 transgene (42) and human clinical studies.

## Data Availability Statement

The raw data supporting the conclusions of this article will be made available by the authors, without undue reservation.

## Author Contributions

Project conception: JW, XL, and LS. Experimental design: JW, XL, DC, LS, ZZ, and LR. Experimental work: XX, CW, YW, XD, and TJ. Data analysis: JW, XL, LR, CDC, XX, and ZZ. Writing original draft: JW, XL, LS, DC, and XX. Writing review and editing: JW, XL, DC, LS, and CC. All authors contributed to the article and approved the submitted version.

## Funding

This work was supported by grants from the National Major Sciences & Technology Project for Control and Prevention of Major Infectious Diseases in China (2018ZX10301401 to XL), the National Natural Science Foundation of China (81930063 and 81971948 to JW and XL), Chinese Academy of Medical Sciences (CAMS) Innovation Fund for Medical Sciences (2016-I2M-1-014 and 2016-I2M-1-005 to JW and XL), National Key R&D Program of China (2020YFA0707600 to XL), and Beijing Municipal Science and Technology Project (Z2011000010200005). LS is supported by Parker B Francis Fellowship and American Lung Association Catalyst Award.

## Conflict of Interest

The authors declare that the research was conducted in the absence of any commercial or financial relationships that could be construed as a potential conflict of interest.
